# *BMC Nursing* reviewer acknowledgement, 2015

**DOI:** 10.1186/s12912-016-0132-z

**Published:** 2016-02-17

**Authors:** James Mockridge

**Affiliations:** BioMed Central, Floor 6, 236 Gray’s Inn Road, London, WC1X 8HB UK

## Abstract

The editors of *BMC Nursing* would like to thank all our reviewers who have contributed to the journal in Volume 14 (2015).

**Mahmoud Al-Hussami**

Jordan

**Arwa Alsaraireh**

Jordan

**Mohamad Al-Tannir**

Saudi Arabia

**Elisa Ambrosi**

Italy

**Sharon Andrew**

UK

**Enoch Anto**

Ghana

**Azizollah Arbabisarjou**

Iran

**Lydia Aziato**

Ghana

**Siv Bäck-Pettersson**

Sweden

**Mohammadkarim Bahadori**

Iran

**Jacqueline Barnes**

UK

**Margaret Barnes**

Australia

**Malcolm Battersby**

Australia

**Samira Behboudi Gandevani**

Iran

**Miriam Bender**

USA

**Kristin Bernard**

USA

**Alice Bernet**

USA

**Ilse Blignault**

Australia

**Terese Bondas**

Norway

**Patrick Brzoska**

Germany

**Cathy Campbell**

USA

**Elisabeth Carlson**

Sweden

**Michael Carter**

USA

**Nancy Carter**

Canada

**Marilyn Cash**

UK

**Bipan Chand**

USA

**Madawa Chandratilake**

Sri Lanka

**Lynn Chenoweth**

Australia

**Teris Cheung**

Hong Kong

**Jeannie Cimiotti**

USA

**SJ Closs**

UK

**Kirsi Coco**

Finland

**Anne Corbett**

UK

**Darin Correll**

USA

**Theodore David Cosco**

UK

**Fiona Cowdell**

UK

**Craig Dale**

Canada

**Teresa Damush**

USA

**Maria Grazia De Marinis**

Italy

**K De Vries**

New Zealand

**Maura Dowling**

Ireland

**Marie Edwards**

Canada

**Maryam Esmaeili**

Iran

**Mohammad Esmaeilpour Bandboni**

Iran

**Philip Esterhuizen**

UK

**Ali Fakhr-Movahedi**

Iran

**Amany Farag**

USA

**Laurie Freeman-Gibb**

Canada

**Gordon Gillespie**

USA

**Mattia Gilmartin**

USA

**Pavithra Godamunne**

Sri Lanka

**Yong-Shian Goh**

Singapore

**Dinah Gould**

UK

**Carolyn Graff**

USA

**Gunne Grankvist**

Sweden

**Carol Grech**

Australia

**Tove Hanssen**

Norway

**Marja Härkänen**

Finland

**Cathy Hebert**

USA

**Amanda Henderson**

Australia

**Halim Hennes**

USA

**Kazem Hosseinzadeh**

Iran

**Suh-Ing Hsieh**

Taiwan

**Tiew Lay Hwa**

Singapore

**Sedigheh Iranmanesh**

Iran

**Anthony F. Jerant**

USA

**Esther Johnstone**

USA

**David Jolley**

UK

**Carolynn Jones**

USA

**Carla Jungquist**

USA

**Mari Kangasniemi**

Finland

**Ayise Karadag**

Turkey

**Anuradhani Kasturiratne**

Sri Lanka

**Amanda Kenny**

Australia

**Fiona Kent**

Australia

**Peter Kevern**

UK

**Evangelos Kontopantelis**

UK

**Christiana Kouta**

Cyprus

**Ingrid Larsson**

Sweden

**Emma Lea**

Australia

**Cindy Lee**

Singapore

**Yuh-Shiow Li**

Taiwan

**Issac Lim**

Singapore

**Jamie Lim**

Singapore

**Violeta Lopez**

Singapore

**Karina Lovell**

UK

**Mahmoud Maharmeh**

Jordan

**Verónica V. Márquez Hernández**

Spain

**Randi Martinsen**

Norway

**Bridie McCarthy**

Ireland

**Deborah McDonald**

USA

**Mick McKeown**

UK

**Fong Meng Kum**

Singapore

**Todd Monroe**

USA

**Randy Moore**

USA

**Karen Morgan**

USA

**Sultan Mosleh**

Jordan

**Reza Negarandeh**

Iran

**Elena Neiterman**

Canada

**Karen Newbigging**

UK

**Patricia Nicholson**

Australia

**Alireza Nikbakht Nasrabadi**

Iran

**Linda O'Mara**

Canada

**Carole Orchard**

Canada

**Areej Othman**

Jordan

**Alvisa Palese**

Italy

**Patrick Palmieri**

USA

**Evridiki Papastavrou**

Cyprus

**Abby Parish**

USA

**Arunasalam Pathmeswaran**

Sri Lanka

**Linda Patrick**

Canada

**Lin Perry**

Australia

**Pammla Petrucka**

Tanzania

**Kathryn Pfaff**

Canada

**Virginia Plummer**

Australia

**Angela Poat**

UK

**David Pulsford**

UK

**Jamal Qaddumi**

Palestine

**Hossein Rafiei**

Iran

**Gillian Ray-Barruel**

Australia

**Cheryl Robinson**

USA

**Gennaro Rocco**

Italy

**Angel Romero-Collado**

Spain

**Jacqueline Ross**

USA

**Tay Rosy**

Singapore

**Natalia Sak-Dankosky**

Finland

**Leena Salminen**

Finland

**Niels Sandholm Larsen**

Denmark

**Charles Scerri**

Malta

**Madrean Schober**

USA

**Margo Sendall**

Australia

**Elham Shakibazadeh**

Iran

**Syed Ilyas Shehnaz**

United Arab Emirates

**Mostafa Shokati**

Iran

**Sarah Smith McAlvin**

USA

**Diantha Soemantri**

Indonesia

**Panayota Sourtzi**

Greece

**Liliana Sousa**

Portugal

**Christina M. Stapelfeldt**

Denmark

**Christine Stirling**

Australia

**Karen Strickland**

UK

**Eileen Sullivan-Marx**

USA

**Tarja Suominen**

Finland

**Claire Surr**

UK

**Georgina Sutherland**

Australia

**Daniela Tartaglini**

Italy

**Richard Taylor**

USA

**Stephen Taylor**

UK

**Deirdre Thornlow**

USA

**Daksha Trivedi**

UK

**Hsiu-Min Tsai**

Taiwan

**Mojtaba Vaismoradi**

UK

**Peter Van Bogaert**

Belgium

**Henriëtte Van Der Roest**

Netherlands

**Praveen Vashist**

India

**Jos Verbeek**

Finland

**Peggy Ward-Smith**

USA

**Susan Waterworth**

New Zealand

**Eila Watson**

UK

**Karen Whittaker**

UK

**Lesley Wilkes**

Australia

**Graham Richard Williamson**

UK

**Kathleen Williamson**

USA

**Vivien Xi Wu**

Singapore

**Kourosh Zarea**

Iran

